# Shedding light on occupational exposure to the sun

**DOI:** 10.2471/BLT.24.020324

**Published:** 2024-03-01

## Abstract

Roughly one in three non-melanoma skin cancer deaths worldwide is associated with working outdoors in the sun. Gary Humphreys reports.

Repon Chowdhury has a particular perspective on occupational exposure to the sun. Chairperson of Labour at Informal Economy – a Bangladeshi, membership-based nongovernmental organization that represents street vendors, waste pickers, agricultural workers and other informal economy workers – he has a lot of contact with people who work outside.

“We spend part of our time making them aware of the occupational health risks they are exposed to, including skin cancer, but many of them are really struggling for survival and have other priorities,” Chowdhury says.

Doctor Belén Mobrici, a generalist with *Mutual Senderos*, a nongovernmental organization working with informal economy workers in Buenos Aires, Argentina, faces similar challenges when talking to street vendors. “They tell me a street vendor has to be in the street,” she says. “Much of the time that means being in the sun.”

While the examples may be extreme, the skin cancer risk faced by Chowdhury and Mobrici’s members is shared by outdoor workers worldwide.

It has long been understood that too much exposure to the sun is harmful, with landmark studies establishing the link between solar radiation and skin cancer dating back to the 1960s and 1970s.

Skin cancers break down into two groups: melanoma and non-melanoma (predominantly basal cell and squamous cell carcinoma); the non-melanoma skin cancers developing in the upper layers of the skin, beyond the melanocyte cells that produce skin pigment.

Because melanomas are more likely than non-melanomas to spread and cause serious illness and death, there is a general perception that the latter are less of a concern. However, non-melanoma skin cancers are far more common, and in absolute terms impose a similar or even larger burden of morbidity and mortality. According to the International Agency for Research on Cancer (IARC), there were a reported 332 000 new melanoma cases in 2022, for 59 000 deaths, compared with over 1.2 million reported non-melanoma cases for 69 000 deaths.

Awareness of the risks posed by sunlight has led to health authorities in many countries developing public information campaigns; examples include Australia’s Cancer Council SunSmart campaign, which was launched in 1981 with a catchy ‘Slip, Slop, Slap’ slogan.

“The aim of the slogan was to educate the public about the risks of excessive sun exposure, and mitigate those risks by slipping on a T shirt, slopping on some sunscreen and slapping on a sunhat,” explains adjunct professor Craig Sinclair, head of prevention at Cancer Council Victoria, a non-profit organization based in the state of Victoria, Australia.

The campaign and those that followed (in 2007, the slogan was updated to ‘Slip, Slop, Slap, Seek, Slide’ to reflect the importance of seeking shade and sliding on sunglasses) are widely credited with contributing to a significant shift in attitudes and behaviours, and a reduction in melanoma rates among younger Australians.

“Not everyone gets to choose how much time they spend in the sun.”

Repon Chowdhury

However, as Sinclair is first to admit, the extent to which people choose to follow such guidance in their private lives is a matter of personal choice. The professional lives of outdoor workers are a different matter, as pointed out by doctor Frank Pega, an epidemiologist and health economist at the World Health Organization (WHO): “People can choose whether or not they go to the beach, but the agricultural worker who needs to put food on the table has to go into the field.”

And once in the field, despite their best efforts to cover up, such workers can be exposed to the sun’s ultraviolet rays for protracted periods. “Farmers who plant or harvest rice are likely exposed to the sun for many hours,” says Pega, “and even if they wear a hat, they’re likely to receive ultraviolet radiation reflected back from water in the flooded fields.”

While clearly presenting a public health challenge, the occupational space also presents significant opportunities for positive change. Pega spells it out: “You can make people aware of the risks and encourage behaviour change, but you can actually oblige employers to reduce these risks through policy and regulation.”

For Pega, the key to getting policy-makers and regulators to pay attention to the issue and decide to do something about it is to generate reliable data regarding the risks incurred and the levels of skin cancer associated with exposure.

“Data are of fundamental importance to raising awareness among workers, policy-makers and employers,” he explains. “They are also vital to establishing baselines and measuring progress towards targets.”

Given the disease burden skin cancers impose (they are one of the most commonly diagnosed cancer groups worldwide, with an estimated 1.6 million new cases a year according to the IARC), they are not well monitored.

The picture with regard to occupational cancer is even less clear, one reason being the challenges faced in separating occupational from non-occupational exposure. “The construction worker who spends all day on the site in shorts is probably going to continue to be exposed,” says Sinclair, who notes that Australia, despite being a high-income country with a robust health information system, does not collect such data.

To improve our understanding of occupational exposure, in 2021 Pega and researchers at the International Labour Organization (ILO) carried out a systematic review and meta-analysis of the effect of occupational exposure to solar ultraviolet radiation on malignant skin melanoma and non-melanoma skin cancer.

The resulting report, which was published by WHO in December 2021, pooled 25 case-control studies with 286 131 participants living in 22 countries, and found evidence indicating that outdoor workers have a 60% increased risk of developing non-melanoma skin cancer, compared with indoor workers. The evidence regarding melanoma was judged insufficient to draw any conclusions.

“Sunlight is the third most burdensome occupational carcinogen.”

Frank Pega

The researchers went on to produce WHO/ILO joint estimates of the burden of non-melanoma skin cancer that can be attributed to occupational exposure to solar ultraviolet radiation, which were published in the November 2023 issue of *Environment International*, a peer-reviewed scientific journal.

“The report provides the first estimates of work-related skin cancer that we have globally,” says Pega, underlining the scope and depth of the data on which the report was based. “We were able to tap into 763 labour force surveys for 96 countries, comprising 166 million observations collected between 1996 and 2021,” he says.

Using that data, the researchers estimated cases of workplace exposure to solar ultraviolet radiation and instances of non-melanoma skin cancer across nearly 200 countries in 2000, 2010 and 2019.

To arrive at their exposure risk results, they combined estimates of the population occupationally exposed (as opposed to generally exposed) to ultraviolet sunlight with the risk ratio for non-melanoma skin cancers from the earlier WHO/ILO systematic review.

Among the report’s key findings was the fact that 1.6 billion people of working age (15 years or older) were exposed to solar ultraviolet radiation while working outdoors in 2019, which is equivalent to 28% of all working-age people.

That same year, almost 19 000 people in 183 countries died from non-melanoma skin cancer due to having worked outdoors in the sun, representing roughly one in three non-melanoma skin cancer deaths worldwide.

“Our findings suggest that sunlight is the third most burdensome occupational carcinogen, behind only asbestos fibres and silica dusts,” Pega says.

Another striking finding was that occupational skin cancer was more widely distributed than expected. “There has long been an assumption that populations in some regions are less affected,” says Pega, “but we found that it is as present in Africa as it is in the Americas, Europe, South-East Asia and the Western Pacific. So, it is really a global health issue.”

At the time of the publication’s release, WHO and the ILO issued a joint statement calling for governments, employers, workers and their representatives to work together in a framework of well-defined rights, responsibilities and duties to reduce the occupational risk of ultraviolet exposure; and ILO Director-General, Gilbert F. Houngbo, said, “Unprotected exposure to solar ultraviolet radiation while working is largely preventable through cost-effective measures.”

Research is required to establish just how cost-effective available measures are. Making workers aware of the risks to which they are exposed is one. These interventions can range from the kind of education and outreach done by Chowdhury and his team of volunteers in Bangladesh, to initiatives based on digital technology offering real-time risk assessment.

A good example of the latter is the SunSmart Global UV app developed by Cancer Council Victoria, in collaboration with WHO, ILO and the World Meteorological Organization. The app provides real-time and forecast ultraviolet radiation levels for locations across Australia and the world from trusted agencies, as well as evidence-based health advice from Cancer Council Victoria regarding the amount of sun protection required.

“Because ultraviolet radiation – the cause of skin damage – can’t be felt, people don’t always realize the risk they are taking,” explains Sinclair. “So giving them a way to calibrate the risk is really helpful.”

Other measures could include introducing labour laws with requirements for protective clothing like broad-brimmed hats and long-sleeved shirts and trousers. As Sinclair points out, such laws already exist in Australia where there is a legal obligation on employers to ensure their outdoor staff work in a safe ultraviolet environment, which includes adequate sun protection, clothing and sunscreen.

Governments also have the option of requiring employers to reduce workers’ risk of getting exposed to solar ultraviolet radiation by organizing shifts outside peak sunlight periods, although, as Pega notes, such measures may be more costly and challenging to implement.

“Unprotected exposure to solar ultraviolet radiation while working is largely preventable.”

Gilbert F. Houngbo

Including skin cancer from occupational sunlight exposure in national lists of occupational diseases would also be helpful, opening the door for the design and implementation of effective workers’ compensation regulation. “Workers’ compensation payouts would not only serve to indemnify injured parties, they would also encourage companies to reduce their financial exposure by investing in prevention,” Pega says.

At the medical level, Pega underlines the importance of improving access to early screening for skin cancer, so the disease can be detected and treated quickly. He stresses that this is especially important for outdoor workers in the informal economy who often fall outside the reach of occupational safety and health policy and practice. “Public health and medical services are particularly important to ensure quality care for these disadvantaged workers,” Pega says.

Unfortunately, as Chowdhury, points out, such medical services are often beyond the reach of workers in the informal economy. “Our members mostly use herbal remedies or other traditional medicines for what they call ‘rashes’, and would not be able to afford to see a doctor even if doctors were available,” he says, “and they certainly have no access to specialists.”

Mobrici raises similar concerns. In Buenos Aires, *Mutual Senderos* goes some way to improving access through participant doctors such as Belén Mobrici who are paid by the government. But she underlines multiple challenges that include a lack of first contact health facilities. “Our primary health care system is very weak here, and the current economic crisis is not helping,” she says.

That leaves both Chowdhury and Mobrici with preventive messaging as their best hope. “Making people aware does make a difference,” says Chowdhury. “The truth is not everyone gets to choose how much time they spend in the sun, but they have a certain amount of control regarding how much they are exposed.”

**Figure:**
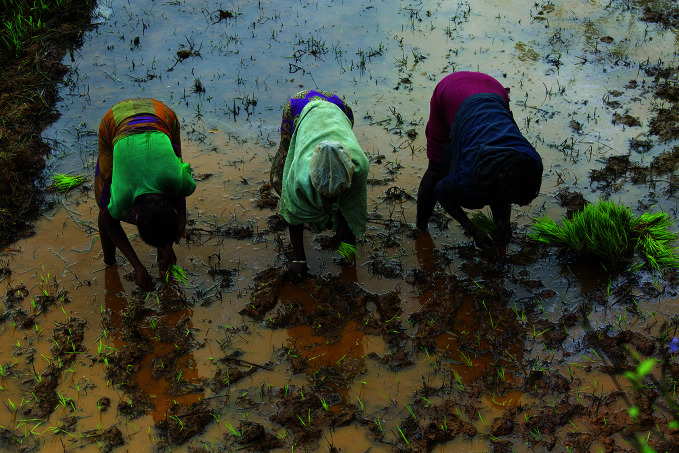
Women plant rice in a field, near Tsiatosika village in Eastern Madagascar

UNICEF/Karel Prinsloo

**Figure:**
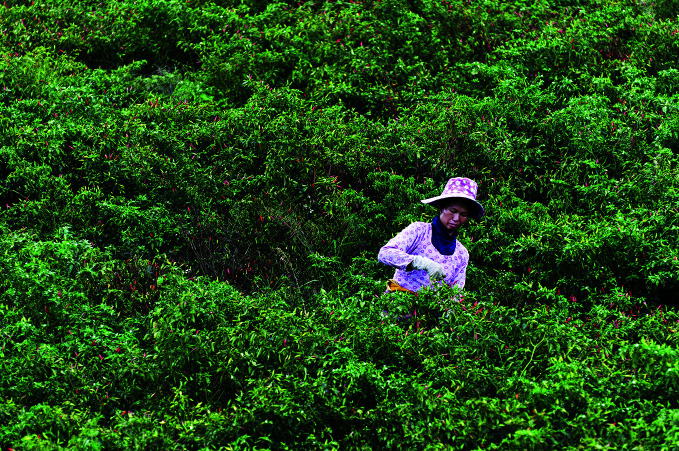
A woman picks chilli peppers in Yunnan Province, China

WHO/Simon Lim

**Figure:**
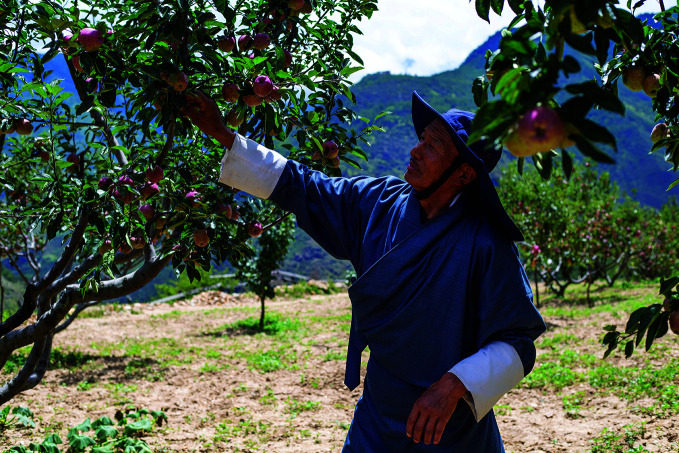
A farmer picks apples in the Chunda Valley, Bhutan

WHO/Sue Price

